# Dr. Barry's Theory of the Venous Circulation

**Published:** 1831-04-01

**Authors:** 


Nothing New under the Sun. Dr. Barry's Theory of the Venous Circulation.

[We received the following anonymous communication by the post.]

To the Editor of the Medico-Chir. Review.

Sir,—I beg leave to refer you to Huxham's work 'de Aëre,' &c., Vol. I., pages 7th and 8th of the Prolegomena, where you will find something respecting the influence of atmospherical pressure on the circulation, tending to shew that Dr. Barry's *discoveries*, on this subject, are not altogether so original as many indulgent critics, notwithstanding their profound erudition, have shown themselves willing to allow. Perhaps on no occasion was it ever more à propos to reiterate the saying of Solomon that, "there is nothing new under the sun." Φιλαλήθης.

---

On referring to Huxham's work, we found, sure enough, Dr. Barry's doctrine laid down in language the most unequivocal, as the following passage will shew.

Huxham.

"Sanguinis ideo momentum adjuvat assidua *atmospheræ* pressura, more velut *antagonistæ* agens contra vim cordis insitam et validè constringentem, quod plus, minusve, omni sphincteri musculo proprium est. Cùm primùm enim, *ope inspirationis*, explicatus pulmo locum dat expellendo è corde sanguini; facto nempe in ductibus pulmonum sanguineis momentaneo *quasi vacuo*, continuo in cor dextrum *impellet sanguinis quantum facile capit pondus atmospheræ, totum corporis habitum comprimens semper*. Eodem porro ipso momento irruens, undeque premens, et elasticus aer haud exiguum addit impetum rapidissimis, per pulmonem currentibus, sanguinis rivulis, ejusque momentum tantum ad auget, ut vel vim cordis sinistri subigere possit: longé enim longeque velocius currit sanguis per pulmones quam per aortæ ramulos." —HUXHAM.

---

We shewed the passage to Dr. Barry, who expressed equal surprise and pleasure at the coincidence — the perfect and complete identity, in fact, of doctrine entertained by Huxham, from reasoning, but proved by Dr. Barry in numerous experiments. This last difference is so great, that we have no hesitation in giving Dr. Barry all the credit of originality—since he declares, and we fully believe him, that he never saw the passage in Huxham, till we shewed him it. We are much obliged to our unknown friend PHILALETHES.

